# Supplementary material for: Proinflammatory Cytokines, but not Glucolipotoxicity, Suppress Nonsense-Mediated RNA Decay to Impair Regulated Transcript Isoform Processing in Pancreatic β-Cells
Source: bioRxiv. 2023 Dec 21:2023.12.20.572623. Preprint. [Version 1] doi: 10.1101/2023.12.20.572623 (PMC10769295; doi:10.1101/2023.12.20.572623)
Supplement: Supplementary file 1 [file media-1.pdf]

Supplementary materials

Proinflammatory Cytokines, but not Glucolipotoxicity, Suppress Nonsense-Mediated RNA Decay to Impair Regulated Transcript Isoform Processing in Pancreatic β-Cells

Seyed. M. Ghiasi[1,2,10†], Piero Marchetti[3], Lorenzo Piemonti[4], Jens H. Nielsen[2], Bo T. Porse[7, 8, 9], Thomas Mandrup-Poulsen[2*] and Guy A. Rutter[1,5,6*]

1. Section of Cell Biology and Functional Genomics, Division of Diabetes, Endocrinology and Metabolism, Department of Metabolism, Digestion and Reproduction, Faculty of Medicine, Imperial College London Du Cane Road, London W12 0NN, United Kingdom
2. Department of Biomedical Sciences, University of Copenhagen, 3 Blegdamsvej, 2200 Copenhagen N, Denmark.
3. Department of Clinical and Experimental Medicine, Islet Cell Laboratory, University of Pisa, 56126, Pisa, Italy
4. Diabetes Research Institute, IRCCS Ospedale San Raffaele, Milano, Italy
5. CHUM Research Centre (CRCHUM), University of Montreal, 900 Rue St. Denis, Montreal, QC, Canada
6. Lee Kong Chian School of Medicine, Nanyang Technological University, 637553, Singapore
7. Biotech Research and Innovation Centre (BRIC), University of Copenhagen, 3 Blegdamsvej, 2200 Copenhagen N, Denmark
8. The Finsen Laboratory, Rigshospitalet, Faculty of Health Sciences, University of Copenhagen, Copenhagen, Denmark
9. Danish Stem Cell Center (DanStem) Faculty of Health Sciences, University of Copenhagen, Copenhagen, Denmark
10. Sanford Burnham Prebys Medical Discovery Institute, La Jolla, CA, USA

[†] Lead investigator: ghiasi@sund.ku.dk, mghiasi@sbpdiscovery.org

*Co-corresponding authors equally contributing to this study: tmpo@sund.ku.dk and g.rutter@imperial.ac.uk

Supplementary Materials and Methods

Materials and Preparations

Recombinant rat and human IL-1β, IFNγ, and rhTNFα were purchased from R&D Systems (Human cytokines: Cat#201-LB-025/CF, 285-IF-100/CF. 210-TA-100/CF, Rat cytokines: Cat# 501-RL-010/CF, 585-IF-100/CF, 510-RT-050/CF, Minneapolis, USA) and tamoxifen from Sigma (Cat# T5648-5G, London, England).

Complete culture media specific for the rat or human β-cell lines and human islets:

For INS-1(832/13) cell pre-culture and experiments RPMI-Glutamax with 11 mM glucose (Cat#61870-010, LifeTechnologies), 10% FBS (Cat# A3840002, LifeTechnologies, Renfrew, England), 1% Penicillin/Streptomycin (P/S; Cat#15140122, LifeTechnologies) and 50 µM 2-mercaptoethanol (Cat#M6250, Sigma).

For human islet preculture RPMI (Cat#11879-020, LifeTechnologies) supplemented with 5.6 mM glucose (Cat#50-152-108, LifeTechnologies), 10% heat-inactivated FBS (Cat#10082147, LifeTechnologies), and 1% P/S; for human islet experiments RPMI (Cat#11879-020, LifeTechnologies) supplemented with 5.6 mM glucose, 2% human serum (Cat#11966025, Cambridge Bioscience; Cambridge, England), and 1% P/S.

For human insulin-producing EndoC-βH3 cells: DMEM (cat #11966025, LifeTechnologies) supplemented with 5.6 mM glucose, 2% BSA-fraction V, 50 µM 2-mercaptoethanol, 10 mM nicotinamide (Cat#N3376, Sigma), 5.5 mg/ml human transferrin (Cat#T3309, Sigma), 6.7 ng/ml sodium selenite (Cat#S5261, Sigma), Penicillin/Streptomycin (Cat#15140122, LifeTechnologies) and Puromycin (Cat#A1113803, LifeTechnologies) (1).

Glucolipotoxic conditions (GLT) were created with 0.5 mM palmitate (cat # P9767, Sigma) conjugated with 0.1% BSA-fraction V (Cat#10735078001, Sigma) + 25 mM glucose as described in (2, 3).

Cell culture, human islet dispersion and treatment

The human EndoC-βH3 and rat INS1(832/13) cell lines were tested negative for *Mycoplasma* and maintained as previously described (1, 4). EndoC-βH3 cells were matured with 1µM of tamoxifen (TAM) for three weeks according to the protocol previously described (1). The TAM-treated EndoC-βH3 cells were seeded onto fibronectin-and ECM-coated culture plates for further experiments (1).

Islets from eight human heart-beating organ-donors (Supplementary Table 1) (>80% purity, donor characteristics listed in Suppl. Table 1A) were isolated under local ethical approval, received in fully anonymous form and pre-cultured as described previously (5). There were no apparent differences in results obtained with islets from male or female donors, and data were therefore combined. For dispersion, 1000 islets were picked after 3 days of pre-incubation, spun down at 1000 rpm for 1 min, washed three times with PBS buffer (w/o Ca^{2+} or Mg^{2+}, Cat# 14190250, Life Technologies), trypsinised with 500 µl Trypsin-EDTA 0,05% (Cat# 25200056; LifeTechnologies) and incubated at 37°C for 3 min. The digested islets were dispersed into single cell suspension by one minute of pipetting. Then, 15 ml complete medium was added to neutralize trypsin, and the dispersed cells were centrifuged at 1500 rpm

for 5 min, re-suspended in the complete medium, mixed by transfection cocktail, re-plated in Fibronectin/ECM-coated 48-well plates and incubated for 48 h.

INS1(832/13), EndoC-βH3 or dispersed human islet cells were seeded in either 6-well plates (1×10^6 cells/well for RNA isolation), 48-well plates (50,000 cells /well for luciferase assay) or 96-well plates (30,000 cells/well for Alamarblue and Caspase-3 activity assays), respectively. After 48h of pre-incubation, INS1 (832/13), EndoC-βH3 or dispersed human islet cells were exposed to cytokine mixture (Cyt) at the concentrations indicated in figures or figure legends for the indicated time points, glucolipotoxic (GLT) conditions, or PBS as negative control.

Luciferase activity assay and NMD activity

Plasmids (650 ng) encoding either human haemoglobin-β (HBB): HBB(NS39 or PTC+) or HBB(wildtype; WT or PTC-) fused with *Renilla (RLuc)*, and *Firefly (FLuc)* plasmid (PMID: 16934750) as transfection efficiency reference were mixed with 250 µl OptiMEM (Cat#31985062, LifeTechnologies), then with 20 µl FUGENE (Cat#E2311, Promega, Southampton, England), vortexed and incubated for 30 minutes at room temperature (RT). Monolayer INS1 (832/13) and EndoC-βH3 cultures were trypsinised into single cells and washed twice with Ca^{2+}/Mg^{2+}-containing PBS buffer (Cat#14080055, LifeTechnologies), whereafter the cell pellets were re-suspended in 2 ml complete medium. One million of either the split INS1(832/13) or EndoC-βH3 cells were added to the transfection cocktail and mixed by pipetting. The transfected cells were seeded in 48-well plates (1×10^5 cells /well) and pre-incubated for 48 h in 37 ºC with 5% CO2.

Renilla and *Firefly* luminescence (*RLuc* and *FLuc*) was measured in a Lumat LB 9507 Tube Luminometer (Berthold Technologies, Bad Wildbad, Germany) by Dual-Luciferase Reporter Assay (Cat# E1910, Promega, Hampshire, England) according to the manufacturer's manual, after lysis of a volume of 20 µl of the transfected cells by adding 100 µl of Passive Lysis Buffer. Luciferase Assay Substrate and Stop&Glo Buffer were manually injected immediately and mixed by 4 times pipetting. The integration time was set on the machine to 1 s after a 4 s delay time. *Renilla (RLuc)* luminescence signals were normalized to the *Firefly (FLuc)* control in both HBB(NS39) and HBB(WT) (6). NMD activity was calculated by dividing the *RLuc/FLuc*-HBB(WT) by the *RLuc/FLuc*-HBB (NS39) as illustrated in supplementary Fig. 1A and originally described (6). Experiments where the control *RLuc/FLuc*-HBB(WT) was affected by Cyt were excluded, so that the resulting NMD activity only denotes the PTC-containing HBB(NS39).

Functional analysis of UPF3A/B overexpression

Expression plasmids (650 ng) encoding UPF3A, UPF3B or UPF3BΔ42 (7) were mixed with 500 µl OptiMEM (Cat#31985062, LifeTechnologies), then with 40 µl FUGENE (Cat# E2311, Promega, Southampton, England), vortexed and incubated for 30 minutes at room temperature (RT). Within the incubation time, the INS1(832/13) and EndoC-βH3 single cells were prepared as above, and one million cells were added to respective transfection cocktail and mixed by pipetting. The transfected cells were re-counted and seeded in 6-well plates (0.5×10^6 cells/well for Western blot analysis of overexpression), 12-well plates (0.3×10^6 cells/well for GSIS) or 96-well plates (0.03×10^6 cells/well for Alamarblue and Caspase 3 activity assays), respectively, and pre-incubated for 48 h at 37ºC with 5% CO_2.

To examine the impact of UPF3A/B overexpression on NMD activity, the transfection cocktail prepared above was divided into two, then 325 ng plasmid of either HBB(NS39) or HBB(WT), and with the

Firefly plasmid were added, followed by vortexing and incubation for 30 min at RT. Five hundred cells of the cell-splits were added as above to the transfection cocktail, mixed by pipetting, seeded in 48-well plates (1×10^5 cells /well) and pre-incubated for 48 hours in 37ºC with 5% CO_2.

Lentiviral shRNA particle production

GPIZ lentiviral shRNA Kits containing shRNA vectors for *Upf2*, *Upf3A* and *Upf3B* genes (Supplementary Table 3) along with a non-silencing shRNA (NS) vector as negative control were from Horizon Discovery (Cambridge, United Kingdom). All plasmids contained a puromycin resistance gene. HEK293FT cells were pre-incubated with OPTI-MEM medium (Cat#11058021, LifeTechnologies) without antibiotics 2h prior to transfection. Lentiviral shRNA particles were produced using the Trans-Lentiviral shRNA Packaging System (Cat#TLP5914, Horizon Discovery, Cambridge, England) according to the manufacturer's instructions. The lentiviral particles were concentrated using Lenti-X™ Concentrator (Cat#631231, Takara, California, USA) according to the manufacturer's instructions. The concentrated virus pellet was dissolved in 1 ml culture medium and stored at -80°C. Multiplicity of infection (MOI) was determined in INS1(832/13) cells using a 10-fold serial dilution along with puromycin-resistance selection (2 µg/ml). After 48h, the MOI of each shRNA lentivirus was scored by counting surviving cells under an inverted fluorescence microscope. Transduced INS1(832/13) and EndoC-βH3 cells were sorted by FACS and reseeded to reach a clonal expansion. The efficiency of knockdown was examined by Western blot analysis.

Library preparation for transcriptome sequencing

Total RNA from the NS control and or UPF2 KD EndoC-βH3 cells treated with cytokines, GLT, or PBS was extracted using TRIZOL (Cat#15596018, LifeTechnologies) with DNase (Cat#EN0521, Life Technologies) treatment and isopropanol precipitation according to the manufacturer's instructions. Quality and quantity of the extracted total RNA from 33 independent biological replicates (i.e., N=6 of each PBS-exposed and or cytokine-exposed NS control and UPF2 KD, and N=4/N=5 of GLT-exposed NS control/UPF2 KD, respectively) were assessed using a NanoDrop-1000 (Thermo Fisher Scientific, Dartford, England). Total RNA (1 µg per isolate) was used as input material for the RNA sample preparations. Sequencing libraries were generated using NEBNext® Ultra TM RNA Library Prep Kit for Illumina® (NEB, Ipswich, USA) following manufacturer's recommendations.

Briefly, mRNA was purified from total RNA using poly-T oligo-attached magnetic beads. Fragmentation was carried out using divalent cations under elevated temperature in NEBNext First Strand Synthesis Reaction Buffer (5X). First strand cDNA was synthesized using random hexamer primer and M-MuLV Reverse Transcriptase (RNase H). Second strand cDNA synthesis was subsequently performed using DNA Polymerase I and RNase H. Remaining overhangs were converted into blunt ends via exonuclease/polymerase activities. After adenylation of 3' ends of DNA fragments, NEBNext Adaptor with hairpin loop structure were ligated to prepare for hybridization. In order to select cDNA fragments of preferentially 150~200 bp in length, the library fragments were purified with AMPure XP system (Beckman Coulter, Beverly, USA). Then 3 µl USER Enzyme (NEB) was used with size-selected, adaptor-ligated cDNA at 37 °C for 15 min followed by 5 min at 95 °C before PCR. Subsequently, PCR was performed with Phusion High-Fidelity DNA polymerase, Universal PCR primers and Index (X) Primer. Finally, PCR products were purified using AMPure XP, and library quality was assessed on the Agilent Bioanalyzer 2100 system (Agilent Technologies, California, USA).

Index codes were added to attribute sequences to each sample. The clustering of the index-coded samples was performed on a cBot Cluster Generation System using PE Cluster Kit cBot-HS (Illumina, San Diego, USA) according to the manufacturer's instructions. After cluster generation, the library preparations were sequenced on an Illumina platform and paired-end reads were generated.

Transcriptomic data analysis

Raw data (raw reads) of FASTQ format were firstly processed through *fastp*. In this step, clean data (clean reads) were obtained by removing reads containing adapter and poly-N sequences and reads with low quality from raw data. All the downstream analyses were based on the clean data with high quality. The RNA-seq clean data were mapped to human reference genome (http://www.ensembl.org/Homo_sapiens/Info/Index) using the Spliced Transcripts Alignment to a Reference (STAR) software. The FeatureCounts algorithm was used to count the read numbers mapped to each gene, then Reads Per Kilobase of exon model per million mapped reads (RPKM) of each gene were calculated based on the length of the gene, and reads count mapped to this gene.

Principal component analysis (PCA), which was used to classify expression patterns according to gene expression level variance, was performed as previously described (8). PCA geometrically projects the high-dimensional dataset onto lower dimensions called principal components (PCs), with the goal of finding the best summary of the data, using a limited number of PCs. The first PC (i.e., PC1) was chosen to minimize the total distance between the data and their projection onto the PC. The second PC (i.e., PC2) was selected similarly, with the additional requirement that it was uncorrelated with PC1(8).

Differential expression analysis *between conditions/groups* (i.e., untreated and cytokines or GLT, or NS control and UPF2 KD (4-6 biological replicates per condition)) was performed using the DESeq2 R package. DESeq2 provides statistical routines for determining differential expression in digital gene expression data using a model based on negative binomial distribution. The resulting p values were adjusted using the Benjamini and Hochberg's approach for controlling the False Discovery Rate (FDR). Genes with an adjusted P value < 0.05 found by DESeq2 were assigned as differentially expressed.

Differential expression analysis of *two conditions* was performed using the *edgeR* R package. The P values were adjusted using the Benjamini and Hochberg method. Corrected P value of 0.005 and |log2 (Fold Change) | of 1 were set as the threshold for significantly differential expression. Gene enrichment analysis (GEA) was performed by biological ontologies including Gene Ontology (GO), Kyoto Encyclopaedia of Genes and Genomes (KEGG), Reactome, Human Disease Ontology (DO) and DisGeNET (https://www.disgenet.org). The *clusterProfiler*, an R package, was used for statistical enrichment of differentially expressed genes, and the gene ontology terms with adjusted P value < 0.05 were considered significant enrichment.

Alternative splicing analysis was performed by the software *rMATS*, that identifies alternative splicing events corresponding to all major types of alternative splicing patterns and calculates the P value and FDR for differential splicing. These types include skipped exons (SE), alternative 5' splice sites (5SS), alternative 3' splice sites (3SS), mutually exclusive exons (MXE), and intron retentions (IR).

cDNA synthesis and RT-qPCR

Purified total RNA (500 ng) was used for cDNA synthesis with the SuperScript™ Kit (Cat#18090010, LifeTechnologies). Real-time Reverse Transcriptase-quantitative PCR (RT-qPCR) was performed on 12

ng cDNA with SybrGreen PCR mastermix (Life Technologies) and specific primers (Supplementary Table 2) and run in a real-time PCR machine (Applied Biosystems QuantStudio, Thermo Fisher Scientific). NormFinder software (9) was used to select the most stable reference gene among Hprt1, Actin and Tubulin. Statistical analysis was carried out on the gene expression levels normalized to the chosen reference gene (see figure legends) through -ΔCt analysis, and figures are presented as logarithmic fold change versus Ctl which was calculated by the -ΔΔCt method.

Western blotting (WB) analysis

Cells were lysed on ice with NP-40 lysis buffer containing protease inhibitor cocktail (LifeTechnologies) and stored at -20°C. Five µg of lysate from each condition of the same experiment adjusted for protein concentration with Bradford assay according to the manufacturer's protocol (BioRad) were loaded in duplicate-quadruplicate on the same gel and were separated by 4-20% SDS-PAGE as appropriate and blotted on PDVF membrane (BioRad). To avoid stripping membranes when staining for multiple proteins, membranes were cut according to the desired molecular weight (MW) range, stained with antibodies against alpha-Tubulin (1:2000) (Cat#T5168, Sigma), UPF2 (1:1000) (LifeTechnologies), UPF3A (1:1000) (Cat#PA5-41904, LifeTechnologies), UPF3B (1:1000) (Cat#PB9843, Boster Bio, Pleasanton, USA) and α-1-antitrypsin (1:1000) (Cat#TA500375, LifeTechnologies), and developed with the chemiluminescence detection system Super Signal (Life Technologies) as previously described (10). High-quality blots of the replicates were quantified relative to the respective loading Tubulin controls. Light emission was captured using an Alphaimager system (Alpha-Innotech, Exeter, England). Band density was quantified using ImageJ software.

Supplementary tables

Table S1. List of human islet donor characteristics

**No.**	**Provider**	**Sex**	**Age**	**BMI**
**1**	**Pisa**	Male	63	29.4
**2**	**Edmonton**	Male	9	18.1
**3**	**Pisa**	Female	64	22.07
**4**	**Milan**	Female	53	27.2
**5**	**Edmonton**	Male	42	29.9
**6**	**Edmonton**	Male	41	33.1
**7**	**Pisa**	Female	76	23.9
**8**	**Pisa**	Male	80	23.03

Table S2. List of specific primers for RT-qPCR. (* taken from (10))

Human genes	Forward	Reverse
ADCY8	CCGAGGGGAGATCTATGTGA	GCTTCTGCCTTTGCCTATTG
APOA1	TGGATGTGCTCAAAGACAGC	AGGCCCTCTGTCTCCTTTTC
APOC3	CTCCCTTCTCAGCTTCATGC	GTCTGACCTCAGGGTCCAAA
ATF3	GTGCCGAAACAAGAAGAAGG	TGGAGTCCTCCCATTCTGAG
B3GNT5	CTGGCTTGGGAAGATCAAAG	CACGATGAACACGACCAATC
BCL11A	GATAAGCCACCTTCCCCTTC	GGGGCATATTCTGCACTCAT
CDH16	CAAGTCATGAGGTGGTGGTG	GATGGTCAGCAGGAAAGAGC
CDH17	GACAATCGACCCACGTTTCT	GCTCCCGTTTTGTTGTTGAT
COL4A1	GAAGGGTGATCCAGGTGAGA	CACCCTTGTCACCTTTTGGT
DDR1	AATGAGGACCCTGAGGGAGT	CCGTCATAGGTGGAGTCGTT
DDR2	GACTTGCACACCCTCCATTT	GAACCGGACAAATCTGGCTA
GPR176	CCCCACAGAACACCATCTCT	GACGGAAGTGTCAGGGACAT
GPRASP1	AAGAGGCCAGTATGGGGACT	CTTCCTCAGCCTCTTGGTTG
GRB10	TCAGGAGGAAGACCAGCAGT	ACCACTTTGCTTGTCCCATC
GRIA4	TACATTGGTGTCAGCGTGGT	CACCTCCAACAATTCGACCT
HES6	CTCCGGGATCCCCAATAC	CAATTTGGGCTGTGGTCAG
HHEX	GCGAGAGACAGGTCAAAACC	AGGGCGAACATTGAGAGCTA
LRRN2	CCCGAGACTGTGATTTCCAT	AAGTTGCTGAGGCCAGAAAA
LSAMP	GAACCGTTCTGGCATCATTT	CACAGTGACATCCGAGGAGA
MET	ACGGTCCAAAGGGAAACTCT	TCAGGCTGGCTCCTATTTGT
MMP2	ACCCAGATGTGGCCAACTAC	CATCGCTCCAGACTTGGAA
NKX6-3	AGGTCAAGGTGTGGTTCCAG	TGTACTCGTCGTCCTCGTTCT
NRXN2	ACTTCCTATGGAGGCCCTGT	GCTGCAGGTAGTCTCCAAGG
PLXDC2	AGATGACCCCATTACCCACA	CTCCTACGGTTGTGGTGGTT
PPP1R1A	CAAGGATCACACCCACAATG	GGGATGCATTCTGCAGTTTT
PXDN	TTGCGACTGGACTCAAACAC	GGCCTTTCACAGTTCAGCTC
RGS9	TGAGCAGTTCTCATCCAACG	GGAAGCTCTGTCGACCTTTG
SERPINA1	GGCTGACACTCACGATGAAA	GTGTCCCCGAAGTTGACAGT
SLC38A4	GGCTTCTTCTGCCACTATGC	GCTTGGAATTTGGAGGATCA
SLC39A5	GGGTGACCTGGAAGAGTCAA	CAGCAGAGCAAACTGACGAG
SLC4A10	GCTTCCCTGATTTGCATCAT	TTCCCTCCATTCCTTCAATG
SYT2	TCACTGTCTGCATCCTGGAG	CGGTGACCACTACCTGGACT
TBX2	GGGACCAGTTCCACAAGCTA	GTGGATGTACATGCGTTTGG
TENM2	TGAAAAGCTGGCTTGTGATG	GACCTGCTTTCTCTCGGATG
TIMP2	GATGCACATCACCCTCTGTG	GTGCCCGTTGATGTTCTTCT
TIMP3	CTGACAGGTCGCGTCTATGA	GGCGTAGTGTTTGGACTGGT
TNR	GTACAGCGGGGATGACTGTT	AGGTACCGTTGGCACATCTC
VTN	GCTGACCAAGAGTCATGCAA	TGTTCATGGACAGTGGCATT
YWHAZ	TTCTTGATCCCCAATGCTTC	AGTTAAGGGCCAGACCCAGT
ATP5B	CCAATTCTAAATGCCCTGGA	TTTGGTTTTGATGGGACCTC
UPF2	AGAGGGTGGAGATGAAGCTG	TTCATGCAAAAATCCATTGC *
UPF3A	ACACAGCGGCAGTGATAAAG	CCTTCTTCCCTCTGTCTTGG *
UPF3B	AGTCGGGACTATCGATGATGA	GAAGCTCAAAAGTGGGGTTG *
sXBP1	CTGAGTCCGAATCAGGTGCAG	ATCCATGGGGAGATGTTCTGG *
usXBP1	CAGCACTCAGACTACGTGCA	ATCCATGGGGAGATGTTCTGG *
ATF4	TACCAGGGGCTCTCCAAATA	GTGGCTGCTGTCTTGTTTT *
CHOP	CAGAACCAGCAGAGGTCACA	AGCTGTGCCACTTTCCTTTC *
BiP	AGGTGGGCAAACAAAGACAT	TCCCTCTTATCCAGGCCATA *
HPRT1	ATG CTG AGG ATT TGG AAA GG	TAA TCC AGC AGG TCA GCA AA *
Actin	AGCGAGCATCCCCCAAAGTT	GGGCACGAAGGCTCATCATT *
TUBB2B	CTTCCAGCTGACCCACTCTC	GTCATACAGGGCCTCGTTGT

Rat genes	Forward	Reverse
Ins-1	GGGGAACGTGGTTTCTTCTAC	CCAGTTGGTAGAGGGAGCAG *
Ins-2	CAGCACCTTTGTGGTTCTCA	CACCTCCAGTGCCAAGGT *
UPF3A	AGCGACCACTGAGAAACACA	CGAAATCTTTTGTCCCTTGG *
UPF3B	GAAGCTTTGAGCAAGGTCGT	CATCAAATCGGTCCCTGAAT *
sXBP1	CTGAGTCCGAATCAGGTGCAG	ATCCATGGGAAGATGTTCTGG *
usXBP1	CAGCACTCAGACTACGTGCG	ATCCATGGGAAGATGTTCTGG *
ATF4	GTTGGTCAGTGCCTCAGACA	CATTCGAAACAGAGCATCGA *
CHOP	CAGCGACAGAGCCAAAATAAC	TGTGGTGGTGTATGAAGATGC *
BiP	CTGGCACTATTGCTGGACTG	CCACCACTTCAAAGACAGCA *
iNOS	CACCACCCTCCTTGTTCAACA	CAATCCACAACTCGCTCCAA *
HPRT1	GCAGACTTTGCTTTCCTT	CCGCTGTCTTTTAGGCTT *
Actin	GTCCCTGTATGCCTCTGGTC	TAATGTCACGCACGATTTCC *

Table S3. List of gene-specific shRNA sequences.

shRNA plasmids			
**Human**	**abbrev**	**catolgue numebr**	**shRNA sequence**
UPF2	U1	RHS4430-200206494	TATTCCTGGAGAGGAGGAC
UPF2	U2	RHS4430-200232044	TTATGTCTATCTTCACTGA
UPF2	U3	RHS4430-200235133	TCAATATTGATCTGGTCCT
UPF2	U4	RHS4430-200237091	TTCTTGATAATCTTCTTGT
UPF2	U5	RHS4430-200237336	TTTCTCTTATCATCTTCCA
UPF3A	A1	RHS4430-200173940	TTAGCTTCCAGTTTCCTGG
UPF3A	A2	RHS4430-200232700	AGGTTTCTAAAAACTTCTT
UPF3A	A3	RHS4430-200240145	TGTTCTTGAGATCTCTCCA
UPF3A	A4	RHS4430-200220879	TTTGGGCAAATTCTTAAAC
UPF3A	A5	RHS4430-200233091	CTATTTTTAATATATTCCA
UPF3A	A6	RHS4430-200241243	TGAGATCTCTCCACATCCC
UPF3B	B1	RHS4430-200196678	TATGTGATGAAGTCCTCAC
UPF3B	B2	RHS4430-200280938	AATCTTCGAATTACCACCT
UPF3B	B3	RHS4430-200281770	TCGCTATCAGAACGCTTGG
UPF3B	B4	RHS4430-200304579	TAAAGTTGATGTATGCTCT
UPF3B	B5	RHS4430-200305947	TTCTTTCTATTTCTCTCCT

Supplementary Figures

Supplementary Figure 1

Supplementary Figure 1

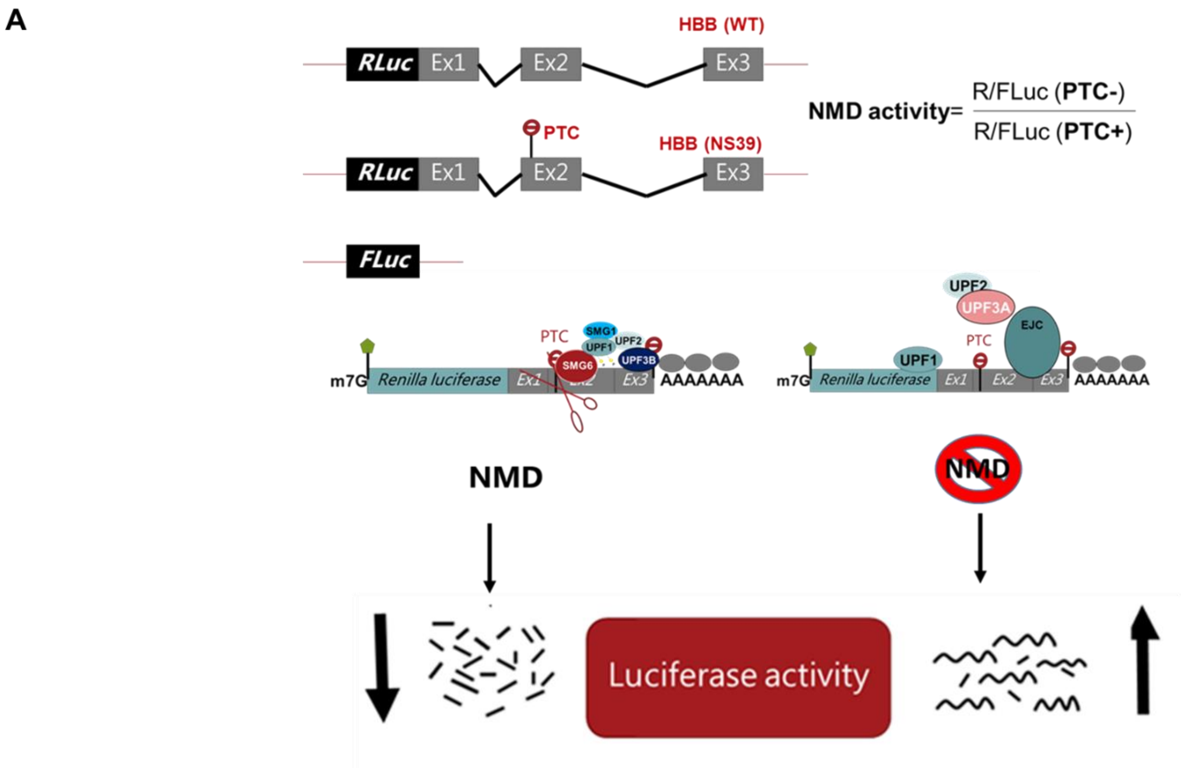

Supplementary Figure 1
B

INS1(832/13)

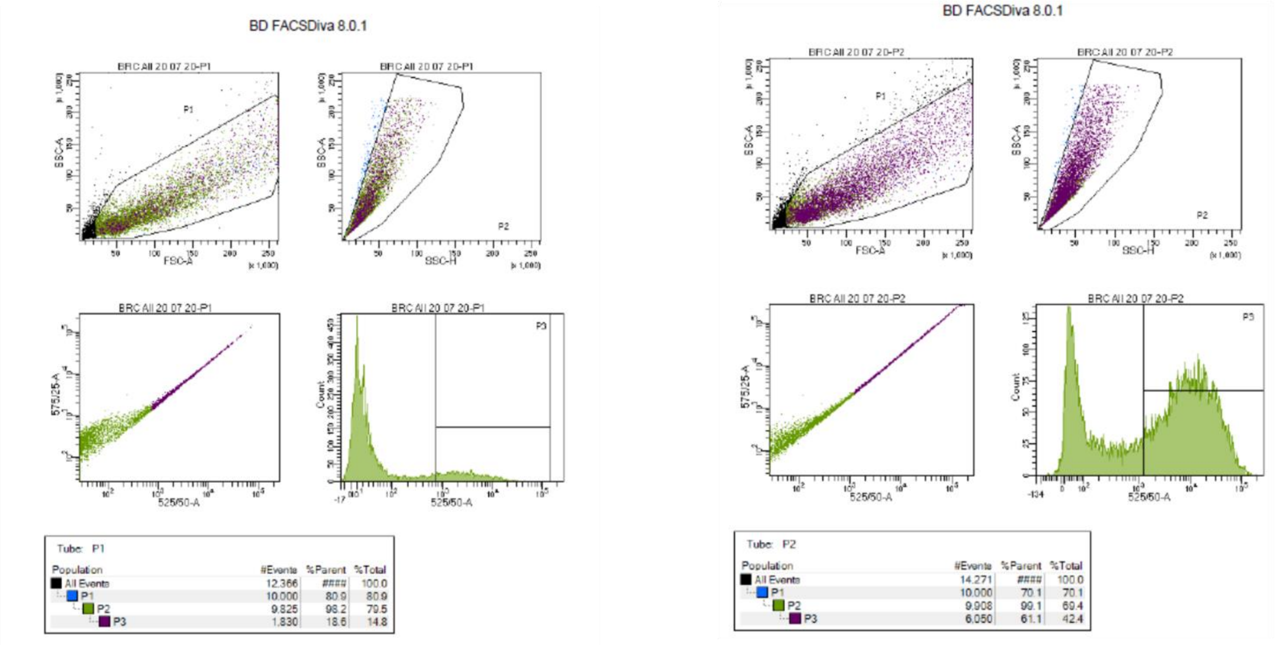

Supplementary Figure 1
C

EndoC-βH3

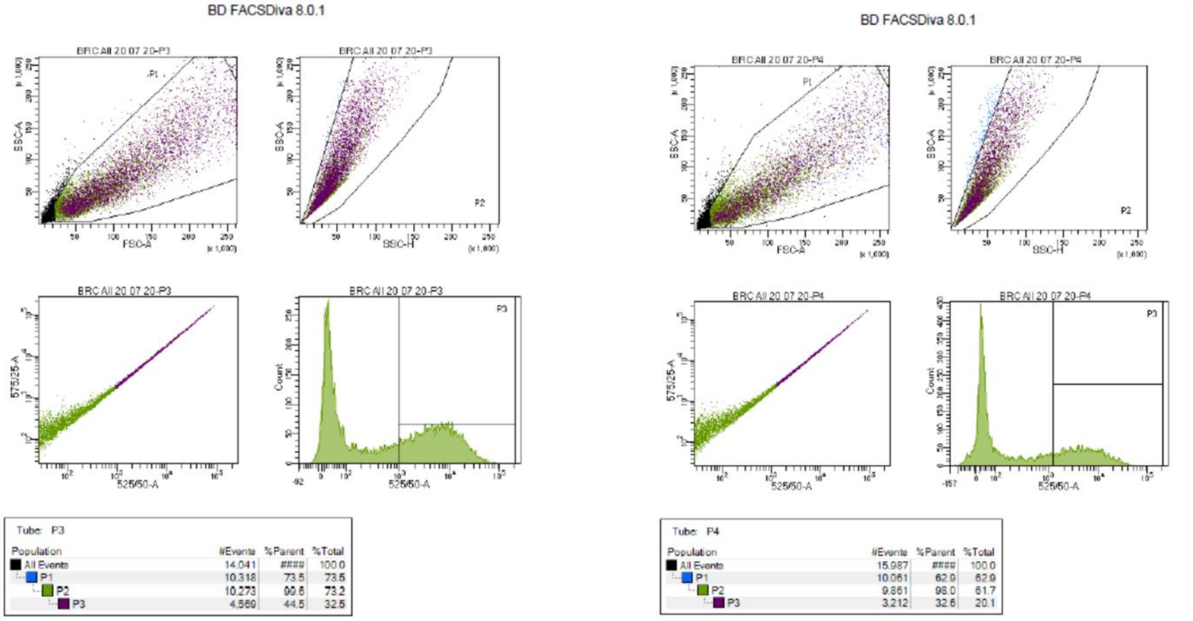

Supplementary Figure 1. Measurement of NMD activity and transfection efficiencies in pancreatic β-cells

A. A schematic representation of luciferase-based NMD activity reporter which expresses either a wild-type (WT or PTC-) or a PTC-containing mutation (NS39 or PTC+) of *Haemoglobin-β (HBB)* gene, designated: HBB(PTC- or WT) and HBB(PTC+ or NS39), respectively, fused to the *Renilla* luciferase (*RLuc*) gene, and an empty plasmid expressing a *Firefly* luciferase (*FLuc*) as transfection control efficiency.

B-C. INS1(832/13) (B), EndoC-βH3 (C) cells were transfected by a GFP expressing plasmid and transfection efficiency was measured by FACS analysis 24 hrs post-transfection as explained in detail in the supplementary methods.

Supplementary Figure 2

Supplementary Figure 2

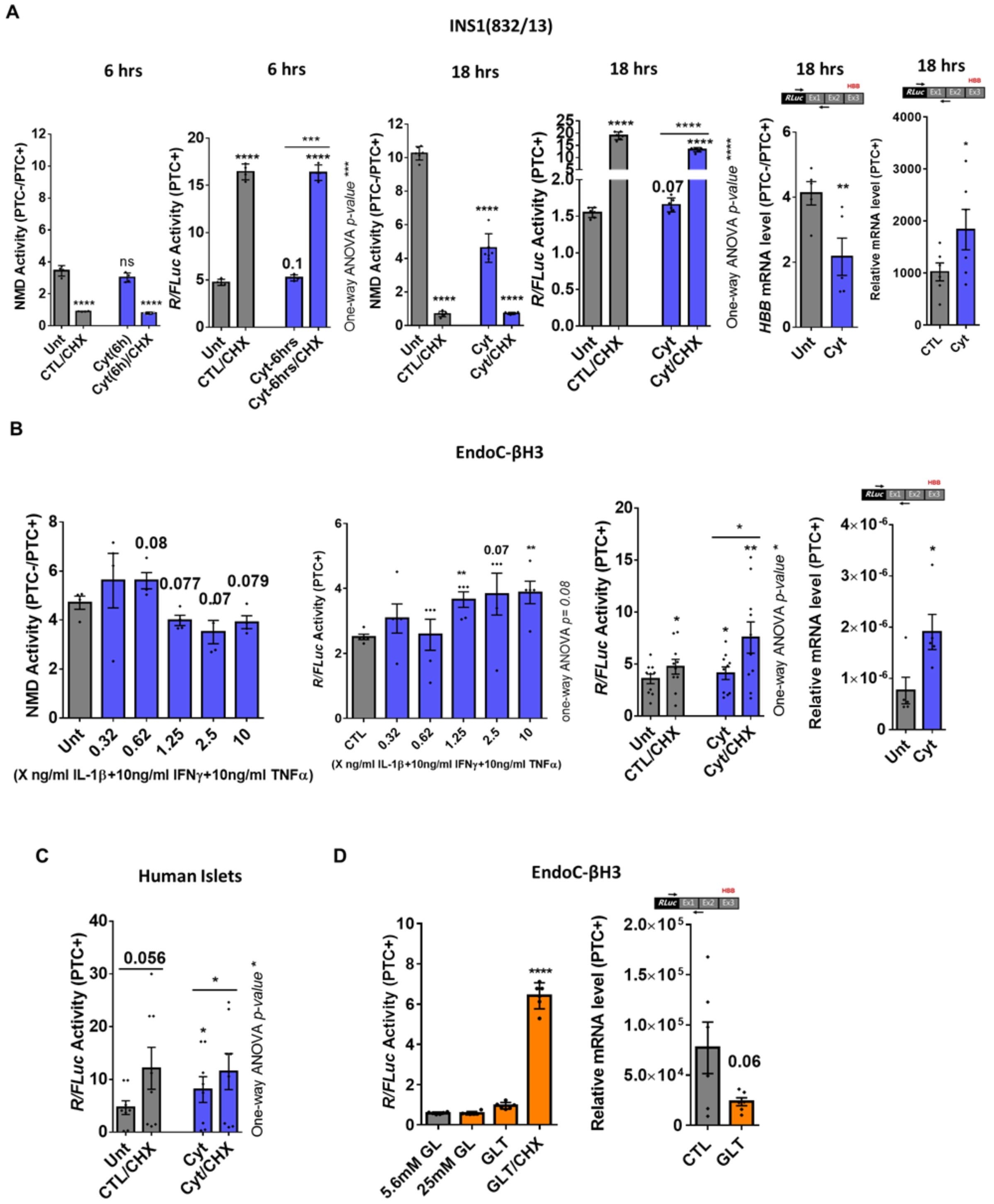

Supplementary Figure 2. Inflammatory cytokines suppress, whereas glucolipotoxicity increases NMD activity in β-cells

A-C. INS1(832/13) (B), EndoC-βH3 (C) cells and dispersed human islet cells (D) were co-transfected with HBB(WT) and or HBB(NS39) and the *Firefly* plasmid and exposed to cytokine combination and or PBS as control (CTL) simultaneously with or without Cycloheximide (CHX) as an inhibitor of NMD activity.

A. Luciferase activity (*R/FLuc* activity of PTC+) was measured in the lysate of the transfected INS1(832/13) cells exposed to cytokine combination (Cyt; 150 pg/mL IL-1β + 0.1 ng/mL IFNγ+0.1 ng/mL TNFα) for 6 hrs and 18 hrs as denoted on top.

B. Luciferase activity (*R/FLuc* activity of PTC+) was measured in the lysate of the transfected EndoC-βH3 cells exposed to cytokine combination (Cyt; X ng/mL IL-1β + 10 ng/mL IFNγ+10 ng/mL TNFα) (right) and (Cyt; 3 ng/mL IL-1β + 10 ng/mL IFNγ+10 ng/mL TNFα) (left) for 18 hrs.

A-B (right). mRNA level of Renilla-HBB(NS39) fused gene and *Firefly* gene in the transfected INS1(832/13) (A-right) and EndoC-βH3 (B-right) cells was quantified by RT-qPCR using specific primers extending the junction of exons 1 and 2 of the *HBB* gene, and *Renilla* gene, or only *Firefly* gene and normalised to actin and tubulin, respectively.

C. Luciferase activity (*R/FLuc* activity of PTC+) was measured in the lysate of the transfected dispersed human islet cells exposed to cytokine combination (Cyt; 3 ng/mL IL-1β + 10 ng/mL IFN-γ+10 ng/mL TNFα) (left) for 18 hrs.

D. EndoC-βH3 cells were co-transfected with HBB(WT) and or HBB(NS39) and the *Firefly* plasmid, and exposed to 5.6 mM glucose, 25 mM glucose (GL), glucolipotoxicity (GLT; 0.5 mM Palmitate+25 mM glucose), and GLT+ Cycloheximide (CHX) for 18 hrs. Luciferase activity (*R/FLuc* activity of PTC+) was measured in the lysate of transfected EndoC-βH3 cells and represented as NMD activity calculated by dividing luciferase activity of HBB(WT)/HBB(NS39) as explained in the methods. D-right. mRNA level of *Renilla*-HBB(NS39) fused gene and *Firefly* gene in the transfected EndoC-βH3 cells was quantified by RT-qPCR using specific primers extending the junction of exons 1 and 2 of the *HBB* gene, and *Renilla* gene, or only *Firefly* gene and normalised to tubulin.

The data are means ± SEM of N=6. The symbol * indicates the Bonferroni-corrected paired t-test values of treatments versus untreated (Unt) (A-B), cytokines (Cyt) that is, otherwise, designated by a line on top of the bars (A-C) or the control (CTL, containing 5.6 mM glucose) (D). * ≤ 0.05, ** ≤ 0.01, *** ≤0.001, **** ≤ 0.0001. ns: non-significant.

Supplementary Figure 3

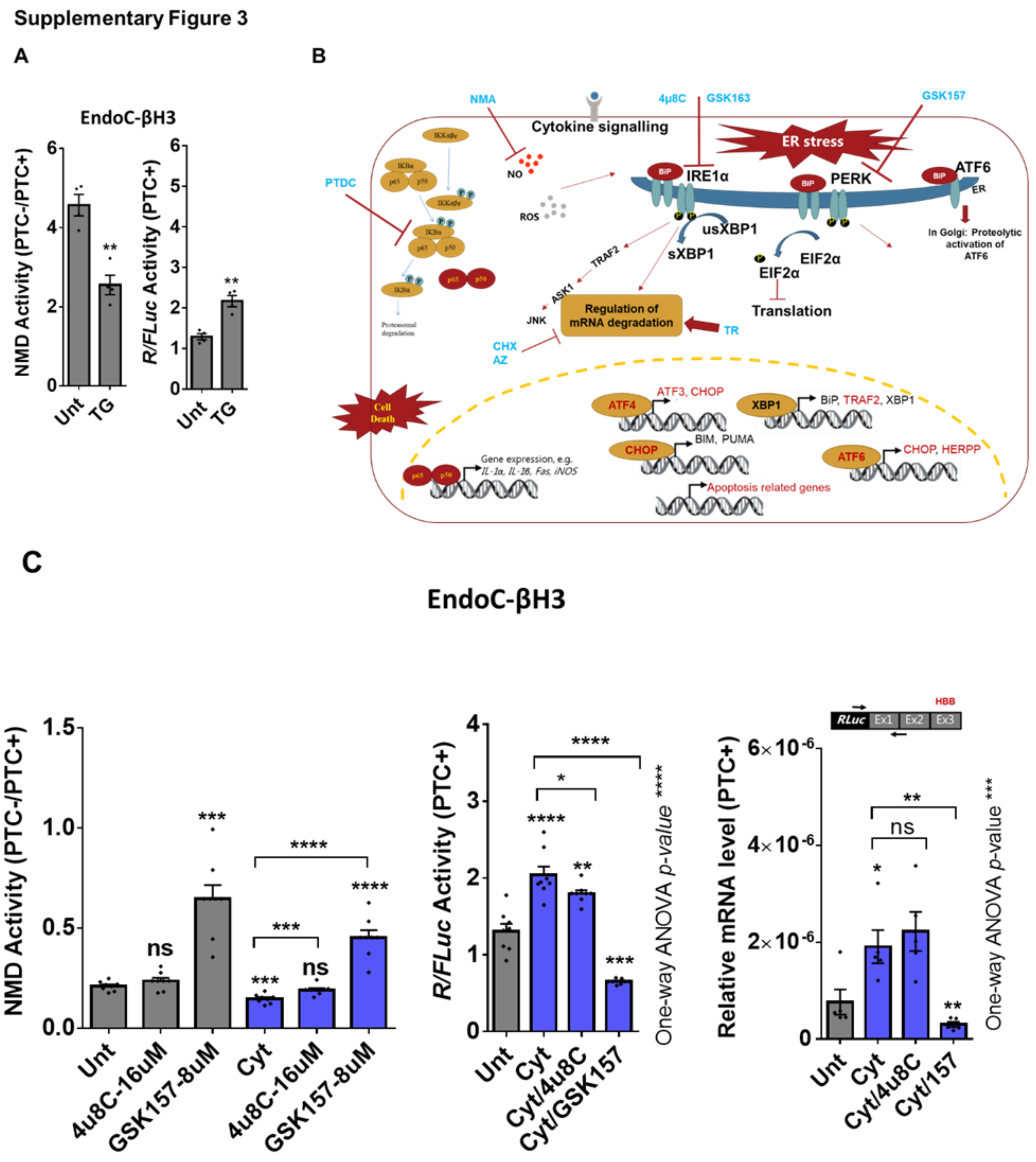

Supplementary Figure 3. ER stress is involved in the inflammatory cytokines-induced suppression of NMD activity in β-cells

A. EndoC-βH3 cells were co-transfected with HBB(WT) and or HBB(NS39) and *Firefly* plasmids and exposed to PBS as control (CTL) or Thapsigargin (TG) or for 18 hrs. Luciferase activity (*R/FLuc* activity of PTC+) was measured in the lysate of EndoC-βH3 cells transfected with HBB(NS39) exposed to PBS as control (CTL) or Thapsigargin (TG), an ER stress inducer as explained in the methods.

B. a scheme of using chemical inhibition of riboendonuclease and kinase activities of IRE1α with 48μC and GSK2850163 (GSK163), respectively, PERK activation with GSK2656157 (GSK157), NF-κB with Ammonium pyrrolidinedithiocarbamate (PDTC) and iNOS with NG-methyl-L-arginine (NMA), and NMD activity with Cycloheximide (CHX) and 5-Azacytidine (AZ), and of NMD activation with Tranilast.

C. EndoC-βH3 cells were co-transfected with HBB(WT) and or HBB(NS39) and *Firefly* plasmids and exposed to PBS as control (CTL), cytokine combination (Cyt; 3 ng/mL IL-1β + 10 ng/mL IFNγ+10 ng/mL TNFα) a lone, and or simultaneously with 16μM of 48μC, an endoribonuclease inhibitor of IRE1α, and or 8μM of GSK2656157 (GSK157) for 18 hrs. Luciferase activity (*R/FLuc* activity of PTC+) was measured in the lysate of the EndoC-βH3 cells as explained in the methods. C-right. mRNA level of *Renilla*-HBB(NS39) fused gene and *Firefly* gene in the EndoC-βH3 cells was quantified by RT-qPCR using specific primers extending the junction of exons 1 and 2 of the *HBB* gene, and *Renilla* gene , or only *Firefly* gene and normalised to tubulin.

The data are means ± SEM of N=6. The symbol * indicates the Bonferroni-corrected paired t-test values of treatments versus untreated (Unt) (A-B) or cytokines (Cyt) that is, otherwise, designated by a line on top of the bars (B). * ≤ 0.05, ** ≤ 0.01, *** ≤0.001, **** ≤ 0.0001.

Supplementary Figure 4

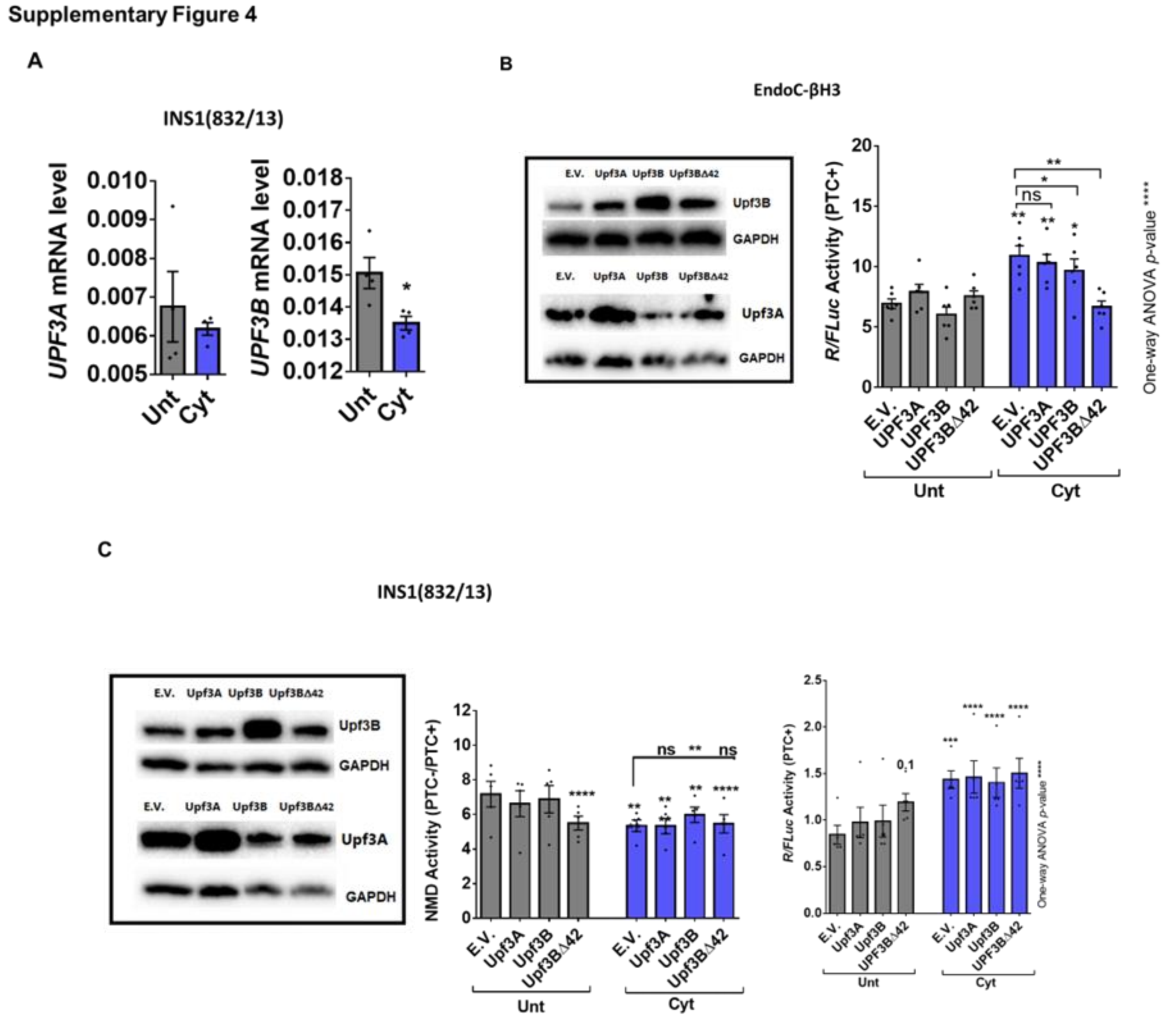

Supplementary figure 4. Inflammatory cytokines-induced suppression of NMD activity is also driven by UPF3B downregulation in β-cells.

INS1(832/13) and EndoC-βH3 cells were co-transfected with empty vector (E.V.), UPF3A, UPF3B and or UPF3BΔ42 (dominant negative of UPF3B) plasmids, and with HBB(WT) and or HBB(NS39), and *Firefly* plasmids and exposed to cytokine combination (Cyt; 150 pg/mL IL-1β + 0.1 ng/mL IFNγ+ 0.1 ng/mL TNFα) and (Cyt; 3 ng/mL IL-1β + 10 ng/mL IFN-γ+10 ng/mL TNFα), respectively, for 18 hrs.

A. mRNA level of *Upf3A* and *Upf3B* genes in INS1(832/13) cells was quantified by RT-qPCR and normalised to actin mRNA.

B. Luciferase activity (*R/FLuc* activity of PTC+) was measured in the lysate of the transfected EndoC-βH3 cells as explained in the methods. The overexpression of Upf3A and Upf3B proteins was examined in the cell lysate (left) by Western blot analysis.

C. Luciferase activity was measured in the lysate of INS1 (812/13) cells transfected with HBB (WT) and represented as NMD activity calculated by dividing luciferase activity of HBB (WT)/HBB (NS39) as explained in the methods. The overexpression of Upf3A and Upf3B proteins was examined in the INS1 (832/13) lysate by Western blot analysis.

The data are means ± SEM of N=6. The symbol * indicates the Bonferroni-corrected paired t-test values of treatments versus untreated E.V. (Unt) (A-B) or cytokines (Cyt)-treated E.V. that is, otherwise, designated by a line on top of the bars. * ≤ 0.05, ** ≤ 0.01, *** ≤ 0.001, **** ≤ 0.0001. ns: non-significant.

Supplementary Figure 5

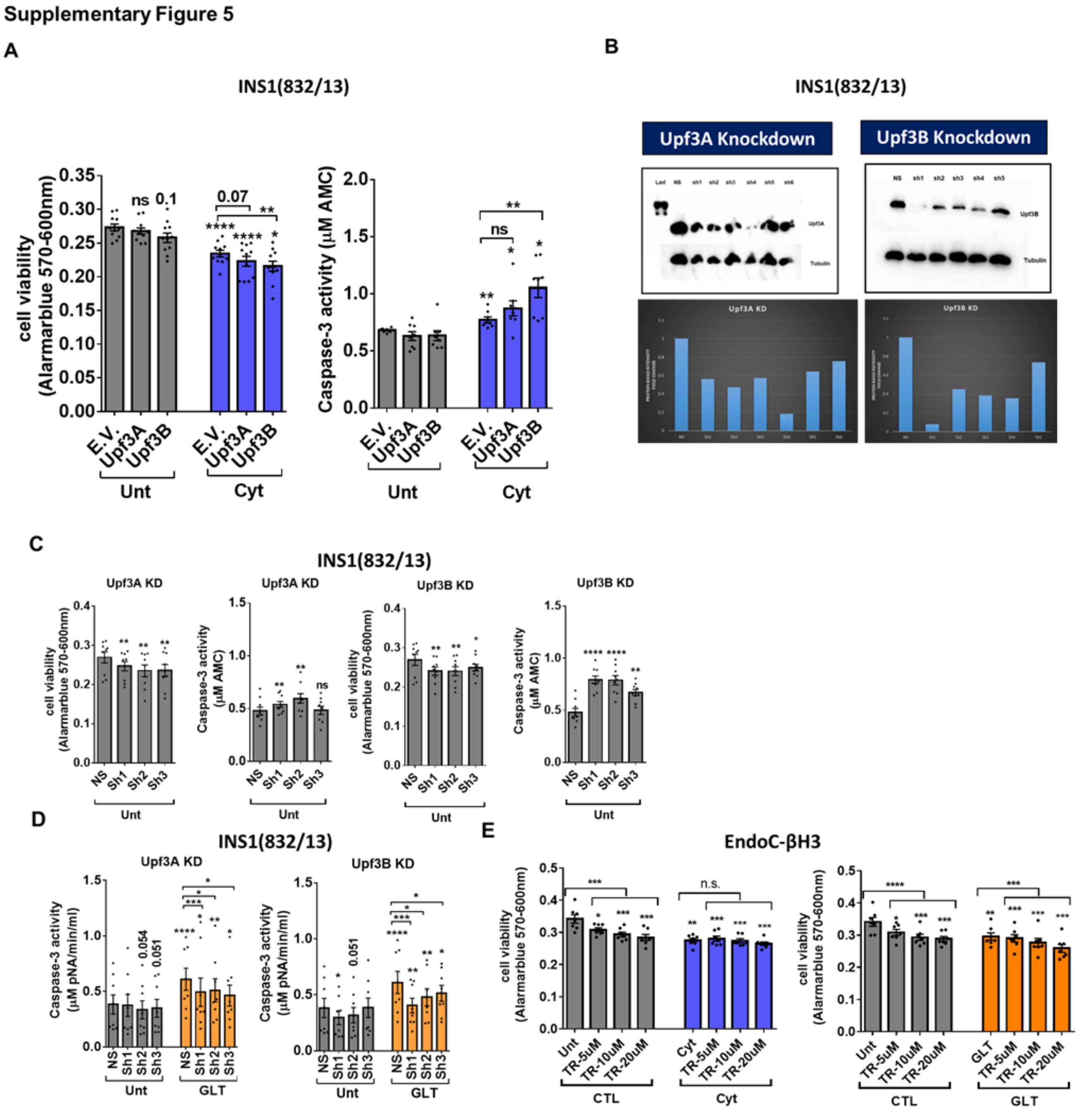

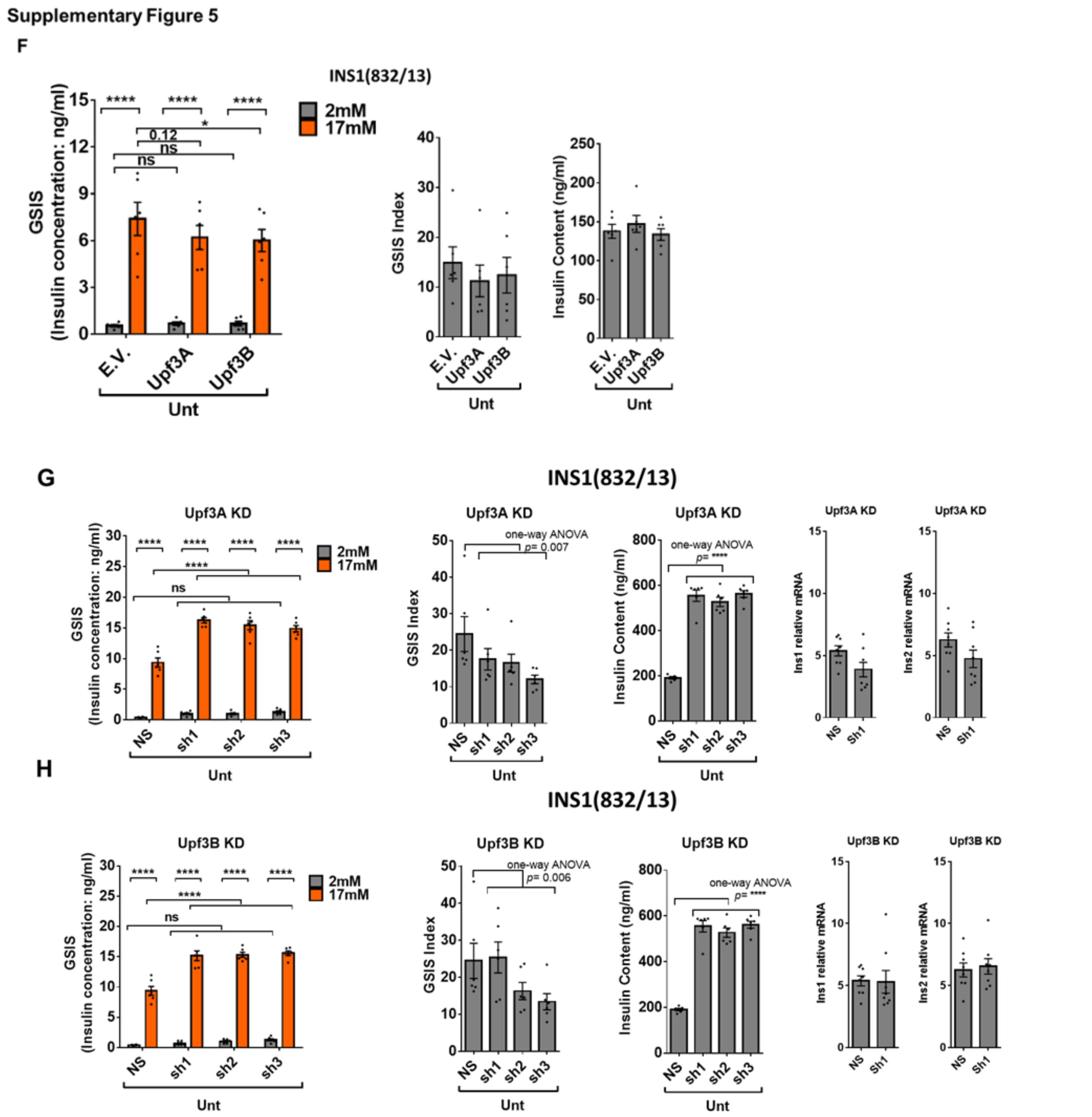

Supplementary figure 5. Upf3A/B knockdown deteriorates basal cell viability associated with increased insulin content, whereas reduces glucolipotoxicity-mediated cell death in INS1 (832/13) cells.

A. INS1(832/13) cells were co-transfected with empty vector (E.V.), Upf3A and or Upf3B plasmids and then, with HBB(WT) and exposed to cytokine combination (Cyt; 150 pg/mL IL-1β + 0.1 ng/mL IFNγ+ 0.1 ng/mL TNFα) for 24 hrs. Cell viability was measured by Alamarblue (left) and caspase-3 activity (μM AMC) (right) assays (N=6).

B-D. INS1(832/13) cell lines with the most efficient stable knock-down of Upf3A and Upf3B (three shRNAs) and non-silencing shRNA as nonsense control (NS) qualified by quantitative WB (B) were treated with PBS, cytokine combination (Cyt; 150 pg/mL IL-1β + 0.1 ng/mL IFNγ+ 0.1 ng/mL TNFα) alone (C) and or glucolipotoxicity (D). The protein band intensity of the WB was quantified using ImageJ and represented as graph (B-bottom). Cell viability was measured by Alamarblue (left) and caspase-3 activity (µM AMC) (right) assays for cytokine effect (C), but only caspase-3 activity for glucolipotoxicity effect (D) (N=6). The caspase-3 activity (µM pNA/min/ml) for glucolipotoxicity effect was measured using colorimetric caspase-3 activity assay kit according to manufacturer's protocol.

E. EndoC-βH3 cells were exposed to cytokine combination (Cyt; 3 ng/mL IL-1β + 10 ng/mL IFNγ+10 ng/mL TNFα) alone or with Tranilast at given concentrations for three days. Cell viability was measured by Alamarblue assay (N=6).

F. INS1(832/13) cells were co-transfected with empty vector (E.V.), Upf3A and or Upf3B plasmids and experimented for glucose-stimulated insulin secretion (GSIS) and insulin contents. Insulin concentration (ng/ml) was measured by insulin ultra-sensitive assay (N=6). GSIS index was calculated by dividing insulin concentration measured in the treatments of 17mM by 2mM glucose.

G-H. INS1(832/13) cell lines with the most efficient stable knock-down of Upf3A (G) and Upf3B (H) (three shRNAs) and non-silencing shRNA as nonsense control (NS) experimented for glucose-stimulated insulin secretion (GSIS) and insulin contents. Insulin concentration (ng/ml) was measured by insulin ultra-sensitive assay (N=6). GSIS index was calculated by dividing insulin concentration measured in the treatments of 17mM by 2mM glucose. The levels of insulin 1 (Ins1) and insulin 2 (Ins2) mRNAs were measured by RT-qPCR and normalised to HPRT1 in the Upf3A (G-right) and Upf3B KD (H-right) cells.

The symbol * indicates the Bonferroni-corrected paired t-test values of treatments versus untreated E.V. (Unt), untreated nonsense control (NS), or cytokines (Cyt)- or GLT-treated conditions that is, otherwise, designated by a line on top of the bars, or corresponding low/high glucose untreated E.V. or NS controls which is designated by lines on the top of the bars (B). * ≤ 0.05, ** ≤ 0.01, *** ≤ 0.001, **** ≤ 0.0001. ns: non-significant.

Supplementary Figure 6

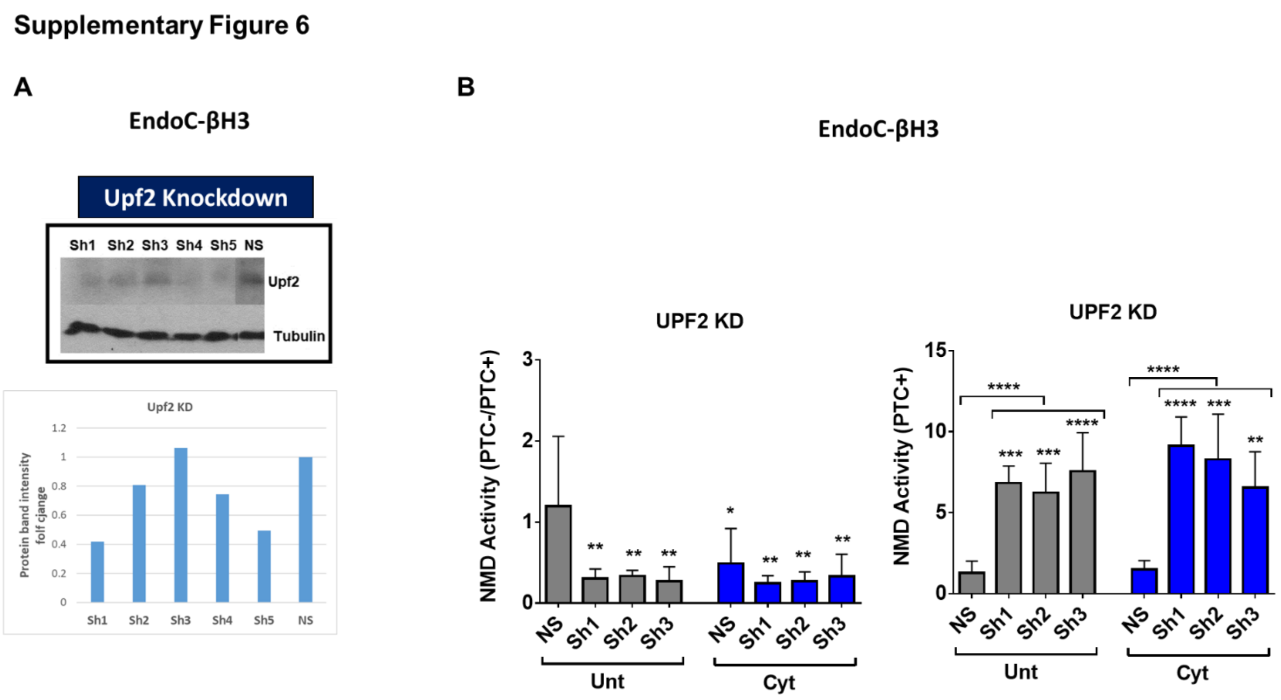

Supplementary figure 6. UPF2 knockdown boosts cytokine suppression of NMD activity in EndoC-βH3 cells

EndoC-βH3 cell lines with the most efficient stable knock-down of UPF2 (three shRNAs) and a non-silencing shRNA as nonsense control (NS) qualified by quantitative WB (A) were co-transfected with HBB(WT) and or HBB(NS39) and *Firefly* plasmids and exposed to PBS as control (CTL), cytokine combination (Cyt; 3 ng/mL IL-1β + 10 ng/mL IFNγ+10 ng/mL TNFα). The protein band intensity of the WB was quantified using ImageJ as represented as graph (bottom). Luciferase activity (B) was measured in the lysate of the transfected cells represented as NMD activity (left) calculated by dividing luciferase activity of HBB(WT)/HBB(NS39) and luciferase activity of HBB(NS39) (*R/FLuc* activity of PTC+; right) as explained in the methods (N=5).

The symbol * indicates the Bonferroni-corrected paired t-test values of treatments versus untreated (Unt) NS control (A-B) or corresponding low/high glucose untreated NS which is, otherwise, designated by lines on the top of the bars (B). * ≤ 0.05, ** ≤ 0.01, *** ≤0.001, **** ≤ 0.0001.

Supplementary Figure 7

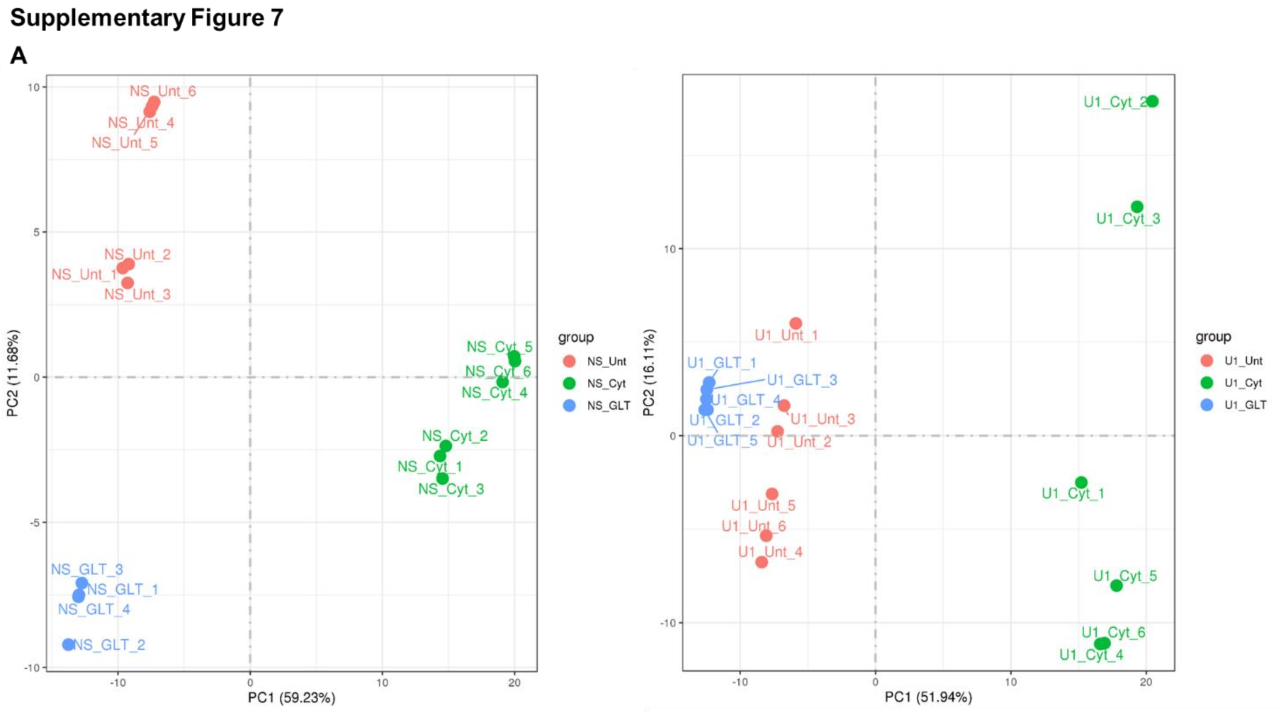

B

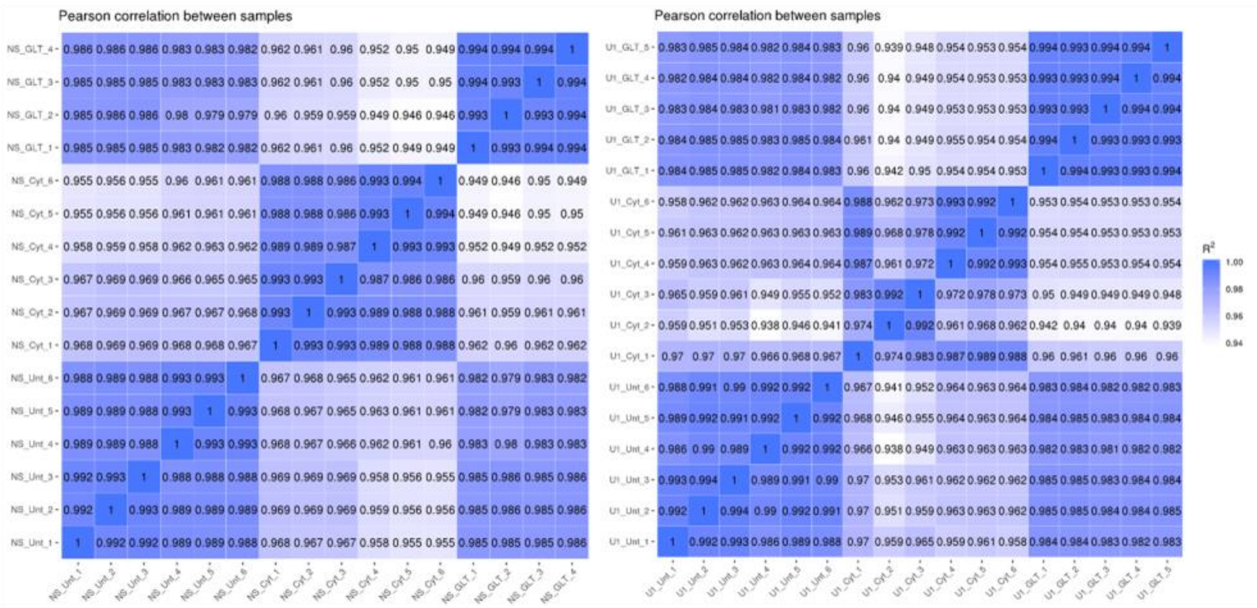
Pearson correlation between samples
Pearson correlation between samples
R²
1.00
0.98
0.96
0.94

C

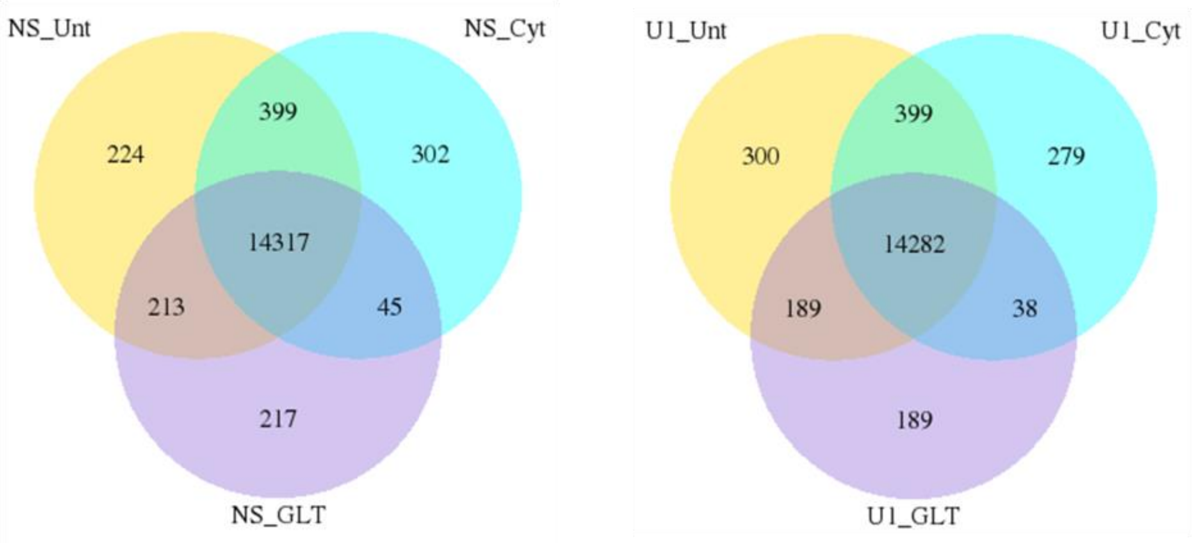
NS_Unt
NS_Cyt
224
399
302
14317
213
45
217
NS_GLT
U1_Unt
U1_Cyt
300
399
279
14282
189
38
189
U1_GLT

D

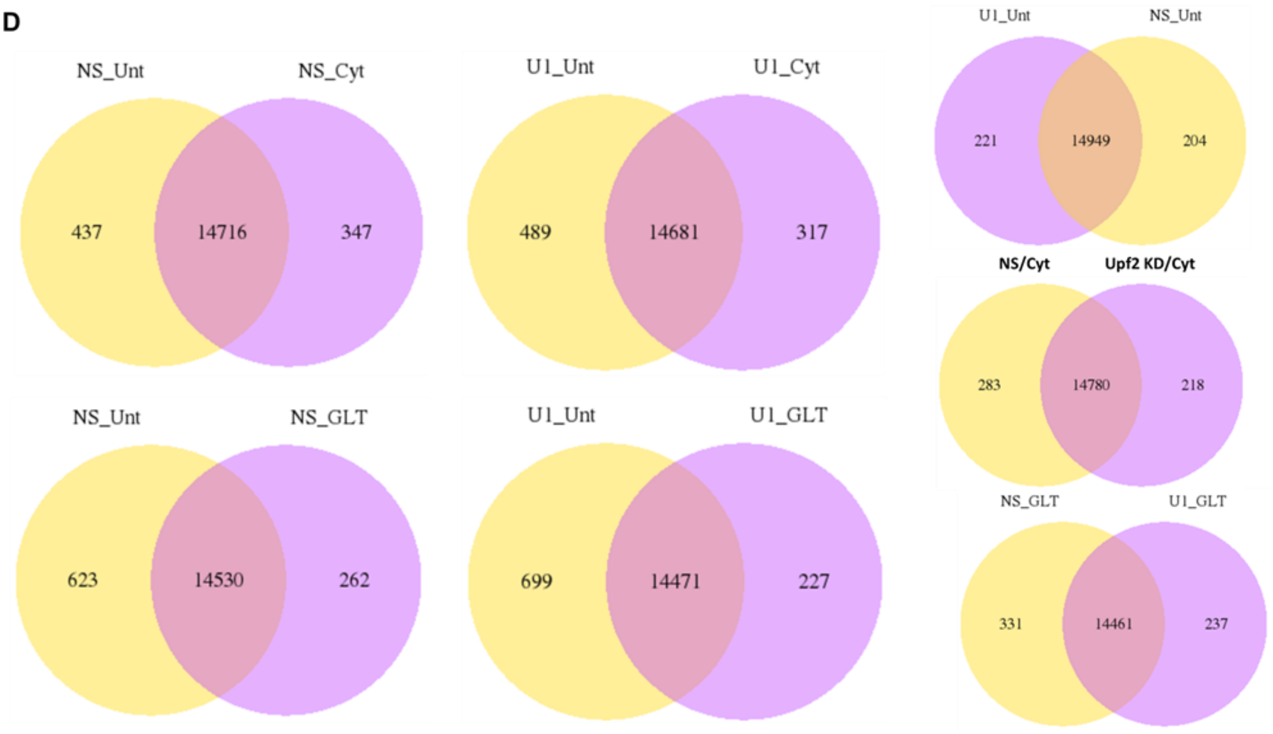
NS_Unt
NS_Cyt
437
14716
347
U1_Unt
U1_Cyt
489
14681
317
U1_Unt
NS_Unt
221
14949
204
NS/Cyt
Upf2 KD/Cyt
283
14780
218
NS_Unt
NS_GLT
623
14530
262
U1_Unt
U1_GLT
699
14471
227
NS_GLT
U1_GLT
331
14461
237

E

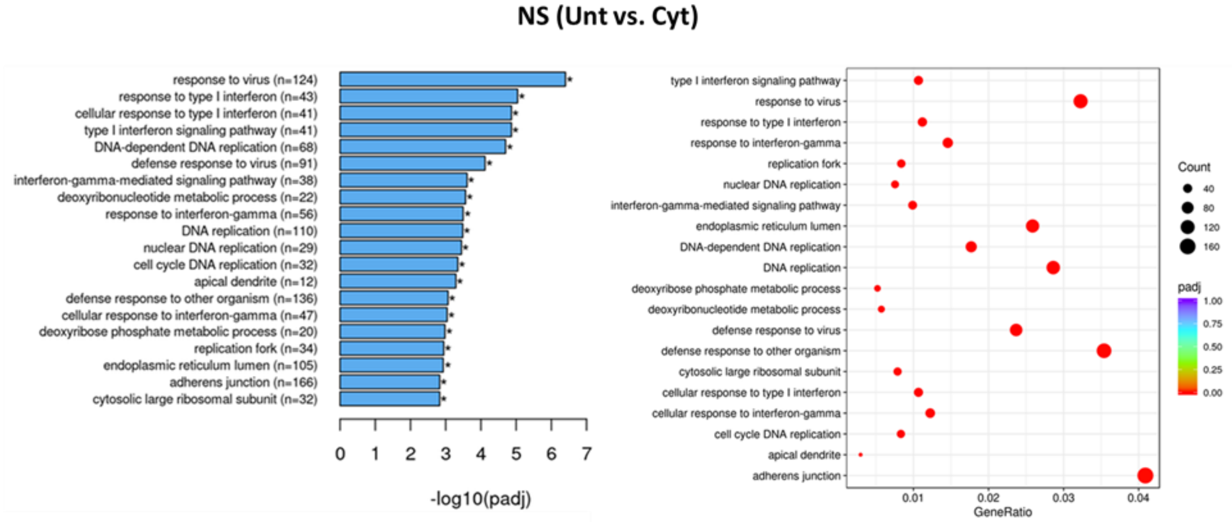
NS (Unt vs. Cyt)
response to virus (n=124)
response to type I interferon (n=43)
cellular response to type I interferon (n=41)
type I interferon signaling pathway (n=41)
DNA-dependent DNA replication (n=68)
defense response to virus (n=91)
interferon-gamma-mediated signaling pathway (n=38)
deoxyribonucleotide metabolic process (n=22)
response to interferon-gamma (n=56)
DNA replication (n=110)
nuclear DNA replication (n=29)
cell cycle DNA replication (n=32)
apical dendrite (n=12)
defense response to other organism (n=136)
cellular response to interferon-gamma (n=47)
deoxyribose phosphate metabolic process (n=20)
replication fork (n=34)
endoplasmic reticulum lumen (n=105)
adherens junction (n=166)
cytosolic large ribosomal subunit (n=32)
0 1 2 3 4 5 6 7
-log10(padj)
type I interferon signaling pathway
response to virus
response to type I interferon
response to interferon-gamma
replication fork
nuclear DNA replication
interferon-gamma-mediated signaling pathway
endoplasmic reticulum lumen
DNA-dependent DNA replication
DNA replication
deoxyribose phosphate metabolic process
deoxyribonucleotide metabolic process
defense response to virus
defense response to other organism
cytosolic large ribosomal subunit
cellular response to type I interferon
cellular response to interferon-gamma
cell cycle DNA replication
apical dendrite
adherens junction
0.01
0.02
0.03
0.04
GeneRatio
Count
40
80
120
160
padj
1.00
0.75
0.50
0.25
0.00

E

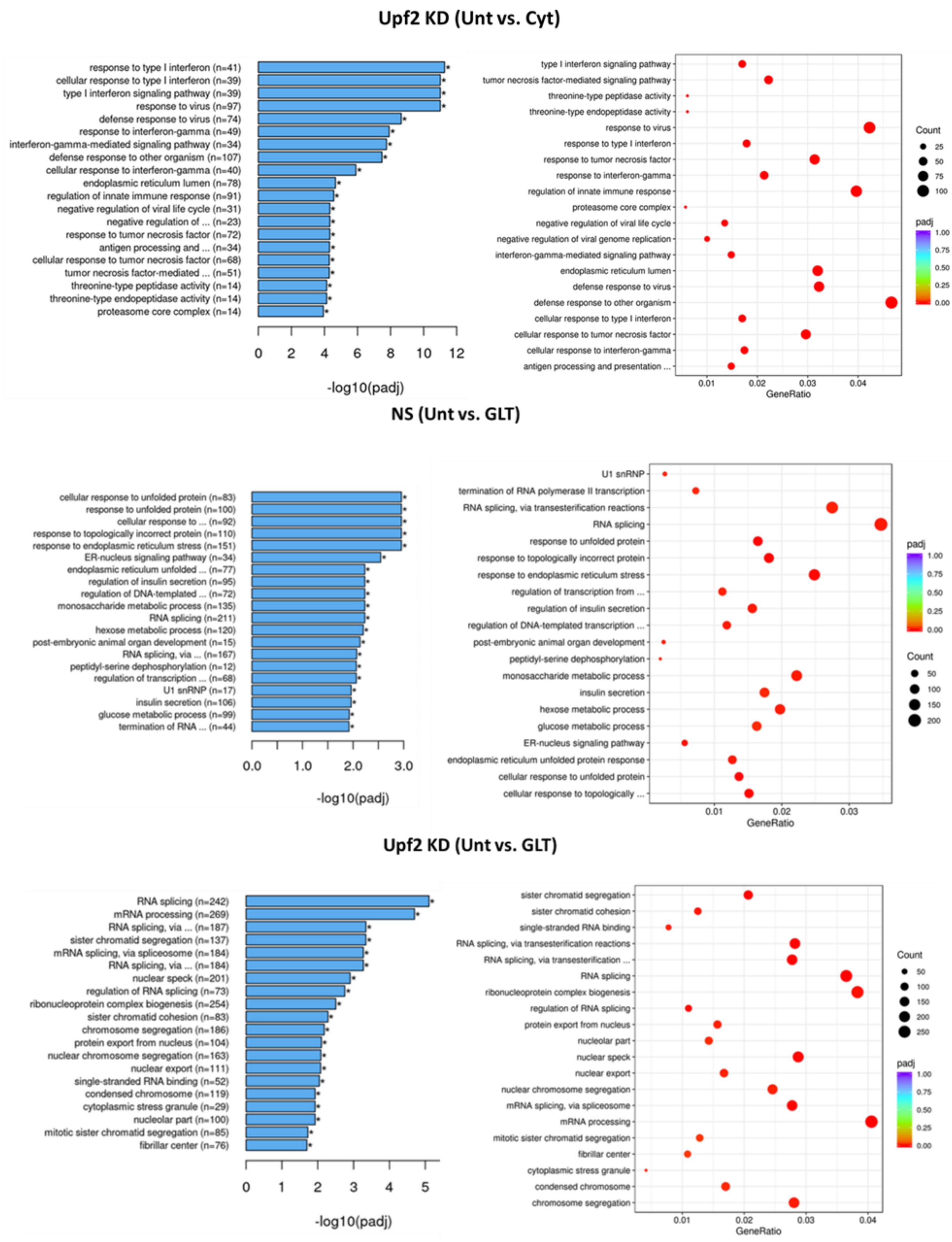

Upf2 KD (Unt vs. Cyt)
response to type I interferon (n=41)
cellular response to type I interferon (n=39)
type I interferon signaling pathway (n=39)
response to virus (n=97)
defense response to virus (n=74)
response to interferon-gamma (n=49)
interferon-gamma-mediated signaling pathway (n=34)
defense response to other organism (n=107)
cellular response to interferon-gamma (n=40)
endoplasmic reticulum lumen (n=78)
regulation of innate immune response (n=91)
negative regulation of viral life cycle (n=31)
negative regulation of ... (n=23)
response to tumor necrosis factor (n=72)
antigen processing and ... (n=34)
cellular response to tumor necrosis factor (n=68)
tumor necrosis factor-mediated ... (n=51)
threonine-type peptidase activity (n=14)
threonine-type endopeptidase activity (n=14)
proteasome core complex (n=14)
0 2 4 6 8 10 12
-log10(padj)
type I interferon signaling pathway
tumor necrosis factor-mediated signaling pathway
threonine-type peptidase activity
threonine-type endopeptidase activity
response to virus
response to type I interferon
response to tumor necrosis factor
response to interferon-gamma
regulation of innate immune response
proteasome core complex
negative regulation of viral life cycle
negative regulation of viral genome replication
interferon-gamma-mediated signaling pathway
endoplasmic reticulum lumen
defense response to virus
defense response to other organism
cellular response to type I interferon
cellular response to tumor necrosis factor
cellular response to interferon-gamma
antigen processing and presentation ...
0.01 0.02 0.03 0.04
GeneRatio
Count
25
50
75
100
padj
1.00
0.75
0.50
0.25
0.00
NS (Unt vs. GLT)
cellular response to unfolded protein (n=83)
response to unfolded protein (n=100)
cellular response to ... (n=92)
response to topologically incorrect protein (n=110)
response to endoplasmic reticulum stress (n=151)
ER-nucleus signaling pathway (n=34)
endoplasmic reticulum unfolded ... (n=77)
regulation of insulin secretion (n=95)
regulation of DNA-templated ... (n=72)
monosaccharide metabolic process (n=135)
RNA splicing (n=211)
hexose metabolic process (n=120)
post-embryonic animal organ development (n=15)
RNA splicing, via ... (n=167)
peptidyl-serine dephosphorylation (n=12)
regulation of transcription ... (n=68)
U1 snRNP (n=17)
insulin secretion (n=106)
glucose metabolic process (n=99)
termination of RNA ... (n=44)
0.0 1.0 2.0 3.0
-log10(padj)
U1 snRNP
termination of RNA polymerase II transcription
RNA splicing, via transesterification reactions
RNA splicing
response to unfolded protein
response to topologically incorrect protein
response to endoplasmic reticulum stress
regulation of transcription from ...
regulation of insulin secretion
regulation of DNA-templated transcription ...
post-embryonic animal organ development
peptidyl-serine dephosphorylation
monosaccharide metabolic process
insulin secretion
hexose metabolic process
glucose metabolic process
ER-nucleus signaling pathway
endoplasmic reticulum unfolded protein response
cellular response to unfolded protein
cellular response to topologically ...
0.01 0.02 0.03
GeneRatio
padj
1.00
0.75
0.50
0.25
0.00
Count
50
100
150
200
Upf2 KD (Unt vs. GLT)
RNA splicing (n=242)
mRNA processing (n=269)
RNA splicing, via ... (n=187)
sister chromatid segregation (n=137)
mRNA splicing, via spliceosome (n=184)
RNA splicing, via ... (n=184)
nuclear speck (n=201)
regulation of RNA splicing (n=73)
ribonucleoprotein complex biogenesis (n=254)
sister chromatid cohesion (n=83)
chromosome segregation (n=186)
protein export from nucleus (n=104)
nuclear chromosome segregation (n=163)
nuclear export (n=111)
single-stranded RNA binding (n=52)
condensed chromosome (n=119)
cytoplasmic stress granule (n=29)
nucleolar part (n=100)
mitotic sister chromatid segregation (n=85)
fibrillar center (n=76)
0 1 2 3 4 5
-log10(padj)
sister chromatid segregation
sister chromatid cohesion
single-stranded RNA binding
RNA splicing, via transesterification reactions
RNA splicing, via transesterification ...
RNA splicing
ribonucleoprotein complex biogenesis
regulation of RNA splicing
protein export from nucleus
nucleolar part
nuclear speck
nuclear export
nuclear chromosome segregation
mRNA splicing, via spliceosome
mRNA processing
mitotic sister chromatid segregation
fibrillar center
cytoplasmic stress granule
condensed chromosome
chromosome segregation
0.01 0.02 0.03 0.04
GeneRatio
Count
50
100
150
200
250
padj
1.00
0.75
0.50
0.25
0.00

Supplementary figure 7. UPF2 knockdown differentially implicates in cytokines and glucolipotoxicity deregulation of EndoC-βH3 transcripts.

EndoC-βH3 cell lines with the most efficient stable knock-down of UPF2 (U1; shRNA-1) and empty vector as nonsense control (NS) were exposed to PBS as control or untreated (CTL or Unt), cytokine combination (Cyt; 3 ng/mL IL-1β + 10 ng/mL IFNγ+10 ng/mL TNFα) and or glucolipotoxicity (GLT; 0.5 mM Palmitate+25 mM glucose). Total RNA was extracted from the treated cells, cDNA library was made and sequenced using Hiseq platform under contracted company's manual. RNA-seq datasets including NS/CTL (N=6), NS/Cyt (N=6), NS/GLT (N=4), U1/CTL (N=6), U1/Cyt (N=6) and U1/GLT (N=5) were analysed through the pipeline described in the methods.

A. Principal component analysis (PCA) of transcripts regulated under Unt, Cyt and or GLT conditions in the samples from NS (left) and UPF2 KD (U1) (right) cell lines.

B. Pearson correlation between the samples from Unt, Cyt and GLT exposed NS (left) and Upf2 KD (U1) (right) cell lines.

C-D. Venn diagram presentation of the number of commonly and differentially regulated transcripts by Cyt or GLT as indicated in the figures.

E. Top GEA-identified transcripts regulated by Cyt compared to untreated in Upf2 KD vs. NS control cells. The expression level is shown as log2 (adjusted p-value <0.05).

Supplementary Figure 8

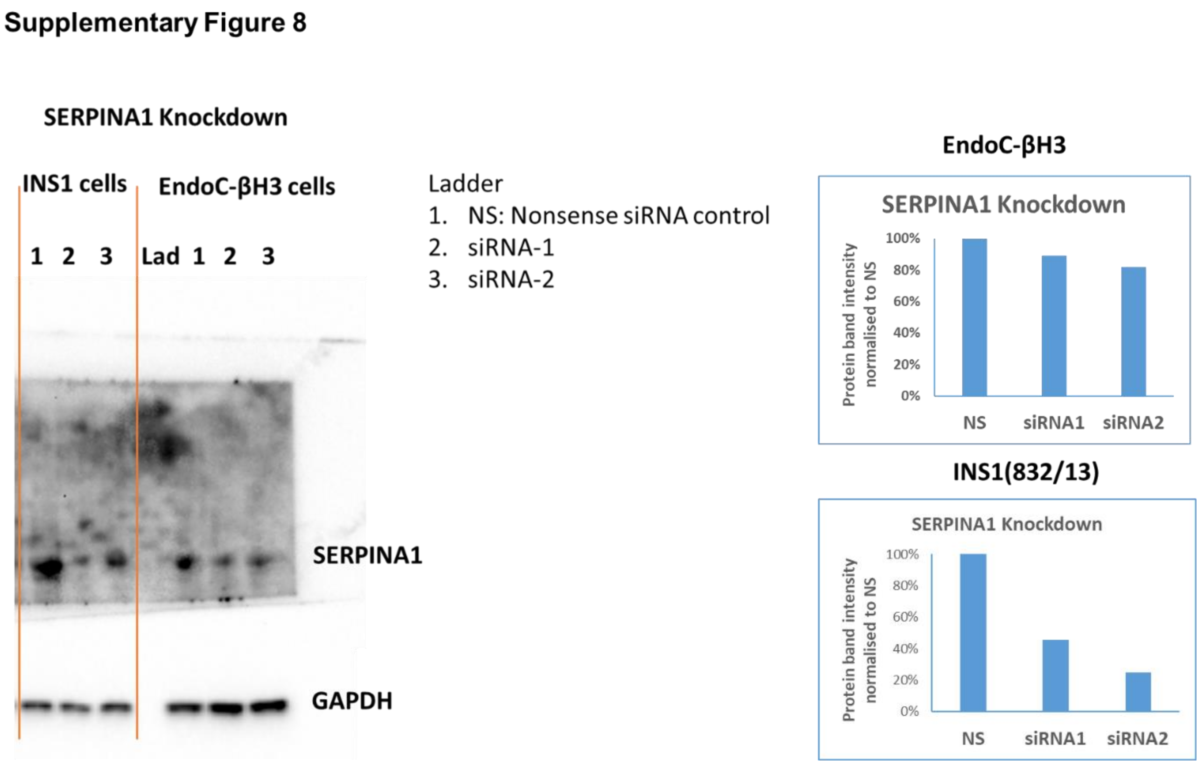

Supplementary Figure 8. SERPINA1 knockdown deteriorates cytokine cytotoxicity for viability and glucose-stimulated insulin secretion index in INS1(832/13) cells.

EndoC-βH3 and INS1(832/13) cells were transfected with siRNAs against SERPINA1 (Si; two species-specific siRNAs for each cell type) and a non-silencing siRNA control (NS), incubated for 24 hours. The knockdown efficiency was checked using quantitative WB as described in the methods.

Supplementary Figure 9

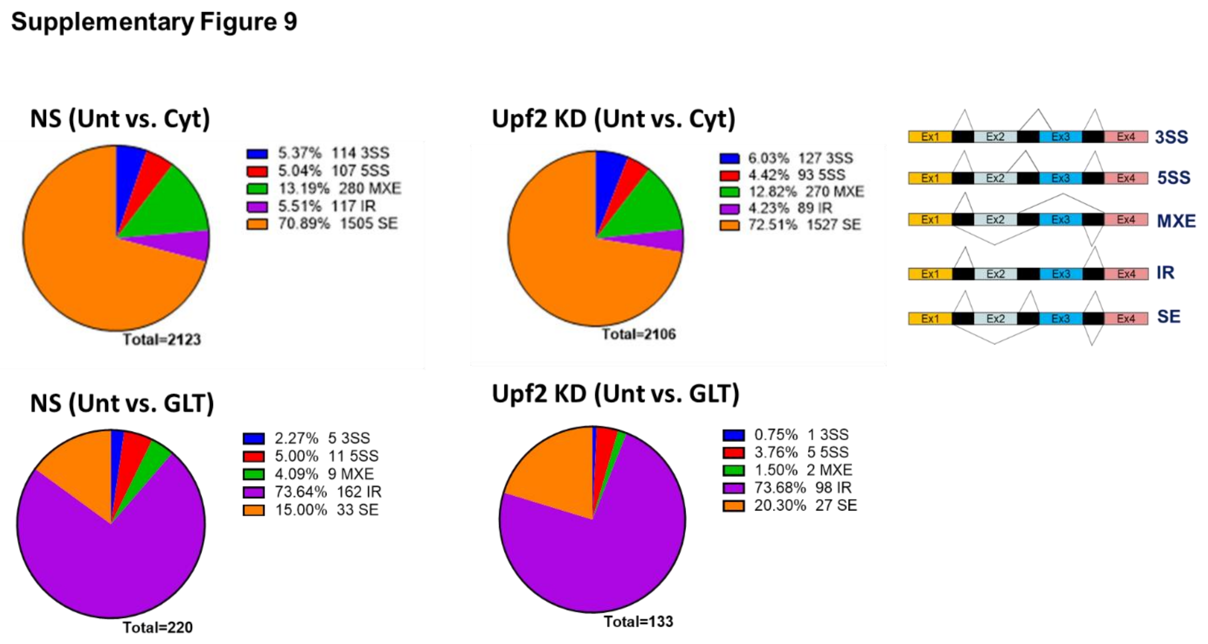

Supplementary Figure 9. UPF2 knockdown potentiates the skipped exon rate of alternatively spliced transcript isoforms increased by cytokines, but not glucolipotoxicity, in EndoC-βH3.

EndoC-βH3 cell lines with the most efficient stable knock-down of UPF2 (U1; shRNA-1) and empty vector as nonsense control (NS) were exposed to PBS as control or untreated (CTL or Unt), cytokine combination (Cyt; 3 ng/mL IL-1β + 10 ng/mL IFNγ+10 ng/mL TNFα) and or glucolipotoxicity (GLT; 0.5 mM Palmitate+25 mM glucose). Total RNA was extracted from the treated cells, cDNA library was made and sequenced using Hiseq platform under contracted company's manual. RNA-seq datasets including NS/CTL (N=6), NS/Cyt (N=6), NS/GLT (N=4), U1/CTL (N=6), U1/Cyt (N=6) and U1/GLT (N=5) were analysed through the pipeline performed by the company (Novogene, Cambridge, England). Alternative splicing events including alternative 3' splice site (3SS), alternative 5' splice site (3SS), mutually exclusive exons (MXE), intron retention (IR) and skipped exon (SE) driven by cytokines or GLT versus untreated in the NS control and Upf2 KD EndoC-βH3 cells were analysed using the software rMATS as described in the methods.

References

1. Benazra M, Lecomte MJ, Colace C, Muller A, Machado C, Pechberty S, et al. A human beta cell line with drug inducible excision of immortalizing transgenes. Mol Metab. 2015;4(12):916-25.
2. Doliba NM, Liu Q, Li C, Chen J, Chen P, Liu C, et al. Accumulation of 3-hydroxytetradecenoic acid: Cause or corollary of glucolipotoxic impairment of pancreatic beta-cell bioenergetics? Mol Metab. 2015;4(12):926-39.
3. Prause M, Christensen DP, Billestrup N, Mandrup-Poulsen T. JNK1 protects against glucolipotoxicity-mediated beta-cell apoptosis. PLoS One. 2014;9(1):e87067.
4. Sekine N, Fasolato C, Pralong WF, Theler JM, Wollheim CB. Glucose-induced insulin secretion in INS-1 cells depends on factors present in fetal calf serum and rat islet-conditioned medium. Diabetes. 1997;46(9):1424-33.
5. Bugliani M, Syed F, Masini M, Marselli L, Suleiman M, Novelli M, et al. Direct effects of rosuvastatin on pancreatic human beta cells. Acta Diabetol. 2013;50(6):983-5.
6. Boelz S, Neu-Yilik G, Gehring NH, Hentze MW, Kulozik AE. A chemiluminescence-based reporter system to monitor nonsense-mediated mRNA decay. Biochem Biophys Res Commun. 2006;349(1):186-91.
7. Neu-Yilik G, Raimondeau E, Eliseev B, Yeramala L, Amthor B, Deniaud A, et al. Dual function of UPF3B in early and late translation termination. EMBO J. 2017;36(20):2968-86.
8. Lever J, Krzywinski M, Altman N. Principal component analysis. Nature Methods. 2017;14(7):641-2.
9. Andersen CL, Jensen JL, Orntoft TF. Normalization of real-time quantitative reverse transcription-PCR data: a model-based variance estimation approach to identify genes suited for normalization, applied to bladder and colon cancer data sets. Cancer Res. 2004;64(15):5245-50.
10. Ghiasi SM, Krogh N, Tyrberg B, Mandrup-Poulsen T. The No-Go and Nonsense-Mediated RNA Decay Pathways Are Regulated by Inflammatory Cytokines in Insulin-Producing Cells and Human Islets and Determine beta-Cell Insulin Biosynthesis and Survival. Diabetes. 2018;67(10):2019-37.
